# Insights into the Classification of Cardiomyopathies: Past, Present, and Future Directions

**DOI:** 10.6061/clinics/2021/e2808

**Published:** 2021-03-19

**Authors:** Vera Maria Cury Salemi, Dania Mohty, Sonia Lages Lustosa de Altavila, Marcelo Dantas Tavares de Melo, Roberto Kalil, Edimar Alcides Bocchi

**Affiliations:** I Unidade Clinica de Insuficiencia Cardiaca, Instituto do Coracao (InCor), Hospital das Clinicas HCFMUSP, Faculdade de Medicina, Universidade de Sao Paulo, Sao Paulo, SP, BR.; II Unidade Coronaria, Hospital Sirio Libanes, Sao Paulo, SP, BR.; III Department of Cardiology, Dupuytren University Hospital, Limoges, France.; IV Heart Center, King Faisal Specialist Hospital & Research Center, Riyadh, Saudi Arabia.; V Universidade Federal da Paraiba, Joao Pessoa, PB, BR.

## INTRODUCTION

A classification system is useful to illustrate the relation between different and complex cardiac diseases. Such a system offers a perspective for understanding a heterogeneous group of diseases based on a logical and systematic standardization. The classification of cardiomyopathies continues to be complex, arbitrary, and challenging. To better understand cardiomyopathy classifications, we provide a critical evaluation from a historical perspective.

### A) History of Cardiomyopathy Classifications

**Figure 1:**
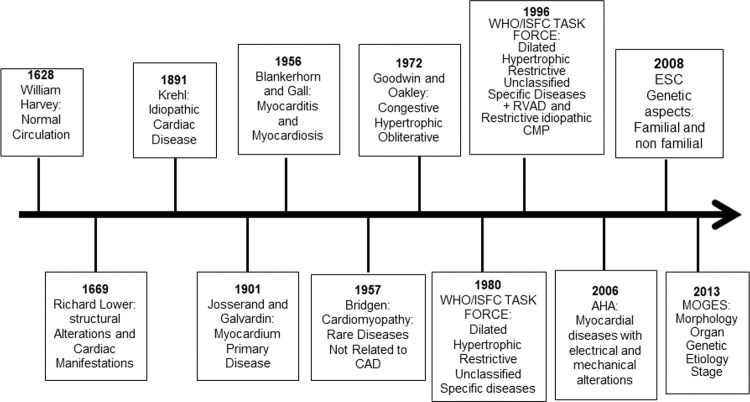
Historical aspects from description of the circulation to cardiomyopathy classification. AHA, American Heart Association; CAD, coronary artery disease; RVAD, right ventricular arrhythmogenic dysplasia; CMP, cardiomyopathy; ESC, European Society of Cardiology; WHO/ISFC, World Health Organization/International Society and Federation of Cardiology.

The description of normal circulation was provided by William Harvey in 1628 in his monograph about the movement of the heart (Figure 1). In 1669, Dr Richard Lower reported cardiac infection and abscess that impaired blood circulation; he also described cardiac dilation in patients with heart failure (1). In the 18^th^ century, heart failure was mostly attributed to valvular heart disease. Furthermore, in the latter part of the 19^th^ century, nonvalvular heart disease was referred to as chronic myocarditis, and it was implied that inflammation was the only cause of the heart disease (2).

In 1891, Krehl described idiopathic diseases of the cardiac muscle, and in 1901, Josserand and Galvardin introduced the term primary myocardial disease (1,2). In 1956, Blankerhorn and Gall described myocarditis as an inflammatory cardiac muscle disease, and myocardiosis as other myocardial diseases. In 1957, for the first time, the name cardiomyopathy was used by Wallace Brigden to refer to uncommon noncoronary myocardial diseases of unknown etiology. In 1961, Goodwin described congestive cardiomyopathy characterized by dilation and heart failure from a different and mostly unknown etiology. In 1968, the term cardiomyopathy was used by the World Health Organization for myocardial disease of unknown etiology, characterized by heart failure and cardiomegaly (2).

Oakley in 1971 described cardiomyopathy as a heart muscle disorder of unknown cause (3). In the same year, John Goodwin suggested classifying primary cardiomyopathy, abandoning the term secondary cardiomyopathy, and classifying cardiomyopathy according to the underlying disease; however, this was complex and did not include all cases. Then, in 1972, Goodwin and Oakley reported cardiomyopathy as a myocardial disease of unknown cause and classified it based on functional pathology findings as congestive, hypertrophic (with or without obstruction), and obliterative cardiomyopathy; however, the last should be classified as a specific heart muscle disease because of its rarity (3).

In 1980, the World Health Organization (WHO)/International Society and Federation of Cardiology (ISFC) Task Force defined cardiomyopathy as heart muscle diseases of unknown etiology, reflecting the poor knowledge of cardiac diseases at that time, and proposed a new cardiomyopathy classification; cardiomyopathies were classified as dilated, hypertrophic, and restrictive, which should be differentiated from unclassified cardiomyopathy that did not fit into these groups. Unclassified cardiomyopathy included latent cardiomyopathy with initial cardiac abnormalities and specific heart muscle diseases of known cause or associated with systemic diseases (4). In addition to systemic or pulmonary hypertension, coronary artery disease (CAD), valvulopathies, and congenital cardiac diseases were excluded.

In 1982, Goodwin stated that “A classification serves to bridge the gap between ignorance and knowledge,” showing the challenges of cardiomyopathy classification at that time. In 1996, the WHO/ISFC Task Force published a new classification based on current knowledge of the dominant pathophysiology, etiology, and/or pathogenesis of cardiac diseases (5). Cardiomyopathy was defined as myocardial disease associated with cardiac dysfunction, and divided into dilated, hypertrophic, and restrictive. For the first time, arrhythmogenic right ventricular cardiomyopathy was included; unclassified cardiomyopathies that did not fit into these groups, such as noncompacted myocardium, mitochondrial, fibroelastosis, and systolic dysfunction with minimal dilation, were included in this classification. Specific cardiomyopathies, previously known by particular heart muscle diseases, that are associated with specific conditions or systemic disorders were included (4). Ischemic, valvular, and hypertensive cardiomyopathies were included in the group of specific cardiomyopathies, leading to confusion about the meaning of myocardial diseases.

### B) Current Cardiomyopathy Classifications

In 2006, the American Heart Association (AHA) published a scientific statement with a new cardiomyopathy classification based on the evolution of genetic testing and diagnostic imaging methods in cardiology. This scientific statement which was designed to facilitate communication between clinicians and researchers, included a description of new cardiac diseases which for the first time included channelopathies and conduction system disorders) (6). The definition of the 2006 AHA classification is that cardiomyopathies are a heterogeneous group of myocardial diseases associated with mechanical and/or electrical dysfunction, frequently with a genetic etiology; they may be systemic or exclusive of the heart. The cardiomyopathies were divided into 1) primary or confined to the heart and divided into genetic, mixed (genetic and nongenetic), and acquired, and 2) secondary, as part of systemic diseases and previously referred to as specific cardiomyopathies.

In 2008, the European Society of Cardiology (ESC) proposed a new definition of cardiomyopathy wherein it was described as a myocardial disease characterized by structurally and functionally abnormal myocardium, because the 2006 AHA classification excluded CAD, hypertension, valvulopathies, and congenital heart disease (7). ESC divided cardiomyopathies into clinically-oriented phenotypes: dilated, hypertrophic, restrictive, arrhythmogenic right ventricular cardiomyopathy, and unclassified. The cardiomyopathies were then subclassified into familial and nonfamilial, where familial is the occurrence in more than one family member or a phenotype that could be caused by the same genetic mutation. Sporadic genetic cardiomyopathy is defined when the mutation occurs for the first time. Nonfamilial is characterized by an absence of relevant family history and is divided into idiopathic or acquired cardiomyopathy. The distinction between cardiomyopathies and specific heart muscle diseases was abandoned. The main criticisms of the 2006 AHA suggested by 2008 ESC classification are: 1) with the understanding of the causes of cardiac diseases improving, the distinction between primary and secondary cardiomyopathies was challenging, 2) primary cardiomyopathy might have systemic symptoms and vice versa, and 3) because channelopathies might not result in morphofunctional phenotypes, it should not be classified as a distinct cardiomyopathy.

In 2013, Arbustini et al. proposed a new classification, similar to the TNM (tumor, node, metastasis) staging system for cancer, known as MOGE(S), where M refers to morphofunctional phenotype, O refers to organ/system involvement, G refers to genetic or familial inheritance pattern, and E refers to etiology and functional status (S) using the American College of Cardiology (ACC)/AHA (A to D) and the New York Heart Association functional classes (I to IV) (8). As per this classification cardiomyopathy is defined as morphological and functional abnormal myocardium in the absence of other diseases that may cause this phenotype. The main advantage of this classification is the global evaluation to improve diagnosis, treatment, and outcomes of cardiomyopathy patients and family; additionally, it facilitates research through a multicenter classification. After genetic evaluation of an index case, a family screening is mandatory to detect family members who may be healthy carriers of the mutation and could develop the disease in the future; they may then be advised to avoid competitive sports or be treated early before cardiovascular deterioration. However, there are still many limitations to MOGE(S), such as the non-inclusion of tachycardiomyopathy, cardiomyopathy associated with endocrine diseases, and peripartum cardiomyopathy in the etiological classification. Furthermore, early stages of myocardial disease and the dynamic evaluation of phenotypes are not embraced; it does not address the risk of sudden cardiac death which is common in these diseases, acute heart failure, and the severity of ventricular dysfunction that could impact the treatment and prognosis of these patients. One major limitation is that Chagas disease, which is a chronic inflammatory cardiomyopathy with specific and severe clinical manifestations, endemic in Latin America and with increasing rates in the United States and Europe due to immigration, is not included. Moreover, coronavirus disease-19 pandemic could cause myocardial damage, and the inclusion of this disease in the current classification is challenging.

### C) Future Directions of Cardiomyopathy Classifications

In the near future, genetic testing will be relevant in clinical practice in association with hybrid cardiac imaging, allowing earlier diagnosis, prognosis, and treatment of patients (9). Genetic testing has many clinical applications and will help address the following issues in daily clinical practice: 1) patients could have a mutation without clinical manifestation, subtle manifestations, or different clinical manifestations for the same mutation; 2) the same phenotype could have a different genetic background, different etiologies, or associations between them (infection, autoimmune, toxic, idiopathic); 3) the myocardial remodeling with change of phenotype, *i.e.*, from hypertrophic to dilated cardiomyopathy with decrease in left ventricular ejection fraction (LVEF), showing the dynamic aspects of cardiomyopathies and impacting treatment strategies; 4) in the early phases of dilated cardiomyopathy with minimal dilation, it is difficult to predict which patient will evolve with a significant dilation; 5) new genetic cardiomyopathies defined recently have shown that the spectrum of dilation, hypertrophy, and restrictive morphologies are dynamic, mixed, and interchangeable (10). We propose a new classification based primarily on genetic testing of the patients and their family, combined with hybrid cardiac imaging; such a multidisciplinary approach could include examining the changes in LVEF, which could impact patient treatment.

## CONCLUSIONS

In conclusion, a critical evaluation of different cardiomyopathy classifications shows that arbitrary and incomplete standardization with some shortcomings and gaps is still present. We believe that in the future, an expert classification based primarily on genetic aspects of the patients and family together with hybrid cardiac imaging and a multidisciplinary approach will allow a better understanding of these complex diseases, especially in patients with mild clinical manifestations, dynamic presentation, or in those with mixed phenotypes.

## AUTHOR CONTRIBUTIONS

Salemi VM wrote the manuscript. Mohty D and de Altavila SLL wrote the original draft of the manuscript. Melo MDT, Kalil Filho R, and Bocchi EA reviewed the manuscript.
